# Primary liver transplantation and liver retransplantation: comparison of health-related quality of life and mental status – a cross-sectional study

**DOI:** 10.1186/s12955-017-0723-8

**Published:** 2017-07-21

**Authors:** Johannes Broschewitz, Georg Wiltberger, Nicco Krezdorn, Felix Krenzien, Julia Förster, Georgi Atanasov, Hans-Michael Hau, Moritz Schmelzle, Andreas Hinz, Michael Bartels, Christian Benzing

**Affiliations:** 1 Department of Visceral, Thoracic, Transplantation and Vascular Surgery, University Hospital Leipzig, Leipzig, Germany; 2 Department of Plastic, Hand and Reconstructive Surgery, Hannover Medical School, Hannover, Germany; 3 Department of Surgery, Campus Virchow and Campus Mitte, Charité-Universitätsmedizin Berlin, Augustenburger Platz 1, 13353 Berlin, Germany; 4 Department of Medical Psychology and Medical Sociology, University Hospital Leipzig, Leipzig, Germany

**Keywords:** Health-related quality of life, Liver retransplantation, SF-36, Hads, Depression, Anxiety

## Abstract

**Background:**

Liver Retransplantation (Re-LT) procedures are associated with an increased risk of morbidity and mortality. Up to date, there is no knowledge on the health-related quality of life and the mental status of these patients.

**Methods:**

Health-Related Quality of Life (HRQoL) was assessed by using the Short Form 36 (SF-36) Health Survey and Mental Status was assessed by using the Hospital Anxiety and Depression Scale (HADS). The patients were examined in different assessments: During regular check-up examinations in the LT outpatient department in 2011 (Survey 1) and in a postal survey in 2013 (Survey 2). Their medical data was collected by using an established database.

**Results:**

We received eligible surveys of 383 patients (55.6%) with a history of LT, of which 15 (3.9%) had undergone Re-LT (Re-LT group). These patients were compared to a group of 60 patients who had undergone only one LT. With regard to their HRQoL, the Re-LT group had significantly lower scores on the scales of physical function (PF, *p* = 0.026), their role-physical (RP, *p* = 0.008), their vitality (VIT, *p =* 0.040), and their role-emotional (RE, *p* = 0.005). The scores for anxiety and depression did not differ significantly between the groups. In a multiple regression analysis, chronic kidney disease was found to be an independent risk factor for decreased scores of PF (*p* = 0.023).

**Conclusions:**

Patients who have to undergo Re-LT procedures are faceing impairments in physical aspects of a HRQoL. Together with clinical results from other studies, the findings of the present examination underline the need for an optimized organ distribution strategy since not all patients listed for Re-LT appear to benefit from it.

## Background

Orthotopic Liver Transplantation (LT) is the only curative treatment for an end-stage liver disease (ESLD), as well as for an acute liver failure (ALF) and a non-resectable hepatocellular carcinoma. This well-established procedure has acceptable long-term outcomes with the overall survival of patients at 71% after 5 years. Nonetheless, graft survival is lower at 64% [1]. Thus, a considerable proportion of patients need to undergo an orthotopic Liver Retransplantation (Re-LT) after their first LT. Today, around 10% of all LT procedures are Re-LTs. Indications for an Re-LT are mostly due to primary nonfunction, venous thrombosis (early graft failure), ischemic type biliary lesions (ITBL) and a recurrence of the initial disease (late graft failure) [2, 3]. For improvements in the overall survival rates raises the likelihood for an Re-LT, as it increases the risks for a chronic graft failure or a recurrence of the primary disease [4, 5].

One of the major problems of Re-LT is the fact that the outcome of these patients is significantly decreased with significantly higher complication and hospitalisation rates compared to recipients of primary LT although Re-LT recipients often benefit from organs with a lower Donor Risk Index. Thus, it is suggested by some authors that a more optimal strategy of distributing organs should be established. This strategy should include restricting their use to patients who obtain a longer term benefit [6].

Besides a mere graft and patient survival, both the Health-Related Quality of Life (HRQoL) and the Mental Status have become an issue of further interest, as they are important outcome factors [7–10]. It is well-known that transplantation improves the HRQoL, as well as the mental status, when compared to those patients on an LT waiting list [7, 11]. However, there are currently only a few examinations that have focused on these particular outcome variables among Re-LT recipients [9, 12–14]. Since Re-LT is associated with an increased postoperative morbidity and a prolonged or insufficient recovery when compared to a primary LT, an impairment of the mental well-being and the HRQoL can be supposed [15].

Thus, the current study has sought to evaluate the HRQoL and the mental status of Re-LT recipients when compared to patients with a history of only one LT. Furthermore, the study aimed to investigate predictors that influence these outcome measures independent of the number of LTs.

## Methods

### Study design

The design of the present study is cross-sectional. It was conducted at the University Hospital of Leipzig, in the Department of Visceral, Transplantation, Thoracic and Vascular Surgery, Germany. It was approved by the Local Ethics Committee (ID: 414–12-17,122,012). All of the surviving patients who had received an LT between 1989 and 2013 were identified. In a second step, those recipients of a double transplant (sequential or simultaneous liver and kidney transplants) and a liverretransplantation were identified.

The patients were recruited in two different assessments. One assessment was performed in 2011. Patients were asked to complete both a written SF-36 health survey and a HADS survey during regular check-up examinations in the LT outpatient department (Survey 1). The second assessment was performed as a postal survey in 2013. Patients received the two surveys, including return envelopes, free of charge, via the mail (Survey 2). In this second assessment, patients were asked to complete the questionnaires within 2 weeks. Those patients who did not answer the first mail were contacted a second time after 2 weeks. They were reminded and asked again to return the questionnaires within another 14 days. The response rate in this second postal survey was 40% [16]. The German version of both questionnaires was used.

The data from both assessments were collected in one database.

### Patients

The inclusion criteria were as follows:

- age > 18 years
- a questionnaire with at least 50% of the questions answered
- their medical records were available

From all of the patients who returned a complete survey, those patients with a history of Re-LT were selected. From the remaining patients with primary LT, a second group was created. The patients were 1:4 matched according to their variables of age and gender. The following two groups were available for further analyses:

- Re-LT recipients (Re-LT group)
- Patients with a history of only one Liver Transplantation (LT-group)

Besides the collection of data on their mental status and their HRQoL, various clinical and demographic variables were included in their analyses. The assessment of comorbidities included arterial hypertension (AH), diabetes mellitus (DM) and chronic kidney failure (CKF). CKF was determined as being a non-reversible reduction of the glomerular filtration rate < 90 ml/min/1.73 m^2^, representing a CKF Stage 2 or higher, as defined by the “Kidney Disease Improving Global Outcomes” (KDIGO) [17]. There was no patient with a dialysis-dependent end-stage renal failure.

### Short form-36 (SF-36)

The SF-36 questionnaire comprises of 36 questions on an individual’s current health status [18]. It is an assessment instrument for the HRQoL of people aged 14 and beyond.

In total, there are 8 different dimensions of HRQoL, which are summarized into two main scales: the physical component summary (PCS) and the mental component summary (MCS).

The 8 dimensions include physical functioning (PF, 10 items), role-physical (RP, 4 items), bodily pain (BP, 2 items), general health perceptions (GH, 6 items), vitality (VIT, 4 items), social functioning (SF, 2 items), role-emotional (RE, 3 items), and mental health (MH, 5 items). The range of possible scores in each subscale is between 0 and 100 points [18, 19].

### Hospital anxiety and depression scale (HADS)

The HADS survey is a tool that is used in order to screen for depression and anxiety. It is a well-established and validated survey that is widely used in the field of medicine [20]. In the present study, we used the German version of the survey, according to Herrmann and colleagues [21].

The questionnaire consists of 14 questions: 7 questions for the assessment of anxiety and 7 questions for the assessment of depression. There are 4 possible multiple-choice answers for each item with a corresponding score of 0 to 3. As a result, the maximum score for each dimension (anxiety and depression, respectively) is 21. A score of 0 to 7 means a normal test result, scores of between 8 to 10 points indicate mild symptoms, and a score of 10 or higher is defined as severe symptoms [22].

### Statistical analyses

The data was collected when using Microsoft Excel (Microsoft, Redmond, USA). A statistics program (SPSS 20.0, IBM, Armonk, USA) was used for the statistical analyses. Before importing the data into the SPSS, all of the questionnaires were digitised and computed (EvaSys© system, Electric Paper Evaluations Systeme GmbH, Lüneburg, Germany).

The continuous variables were analysed by using non-parametric statistical tests (Kruskal-Wallis test, the Mann-Whitney U test and Wilcoxon test). The categorical variables were tested by using the Fisher’s exact test. Multiple regression analysis was performed using a linear regression model. All *p*-values <0.05 were indicated as being of a statistical significance.

*Note: This study has partially analysed data of a large cross-sectional study of transplant candidates and transplant recipients. In parts, it has been previously published* [7, 8, 23, 24]*. However, these publications considered different inclusion criteria and used a different patient subset (e.g. waitlisted patient vs. transplant recipients). In this present analysis, we have reported on the recipients of a Liver retransplantation for the first time. Moreover, the current study has included a second set of patients that were examined earlier (in 2011). So far, this data is unpublished.*

## Results

### Baseline characteristics

**Fig. 1:**
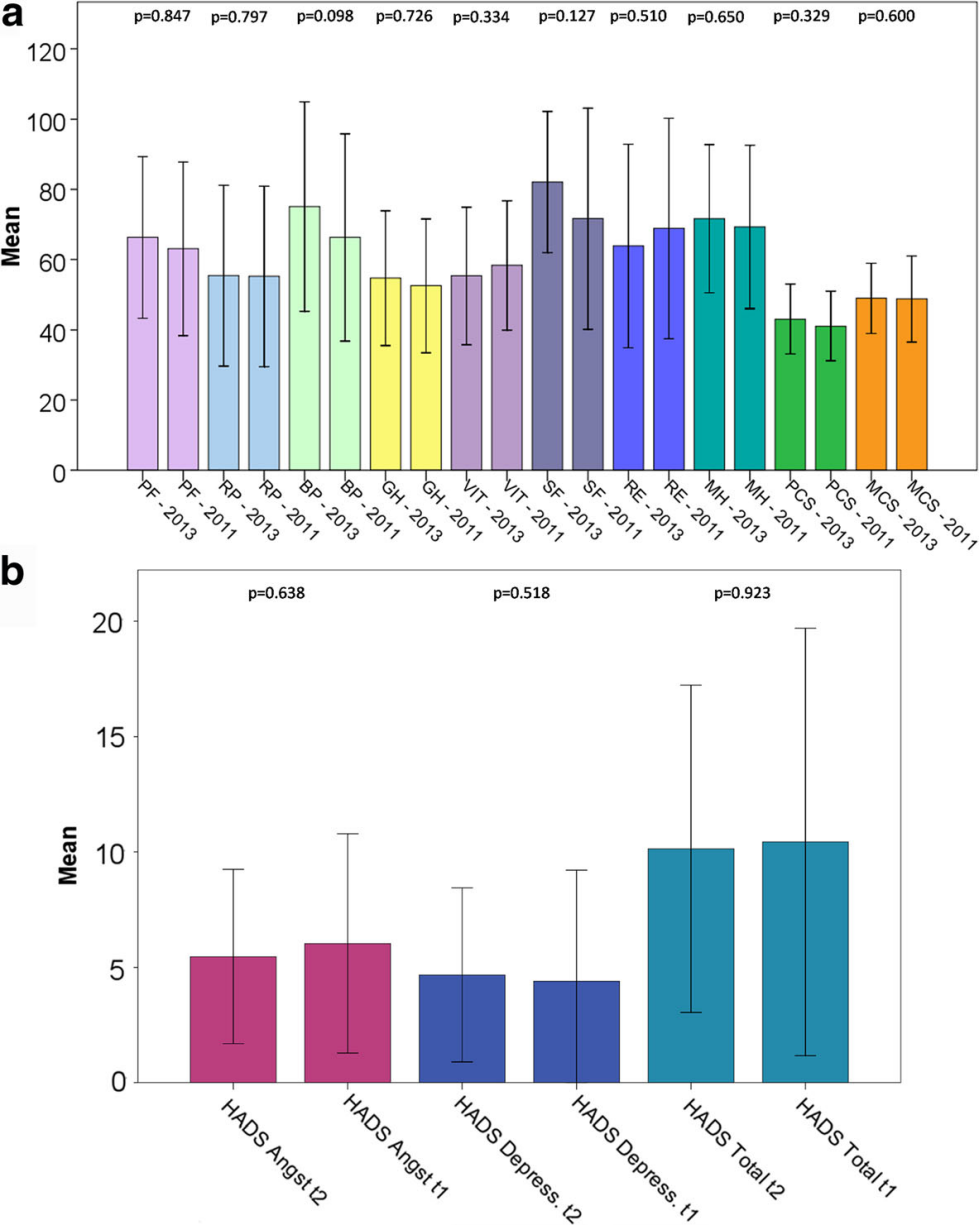
SF-36 (**a**) and HADS (**b**) of the patient group that was interviewed both in 2011 and 2013 (*n* = 33). PF = physical functioning, RP = role-physical, BP = bodily pain, GH = general health perceptions, VIT = Vitality, SF = social functioning, RE = role-emotional, MH = mental health, PCS = physical component summary, MCS = mental component summary

The number of surviving patients who had received an LT was 689. We received eligible surveys from 383 patients (55.6%). Of these 383 patients, 106 (27.7%) were interviewed during Survey 1 and 117 (30.5%) were interviewed during Survey 2. The remaining 160 patients (41.8%) were interviewed twice (in 2011 and in 2013, respectively). For these cases, results from both surveys were statistically compared. The results are shown in Fig. 1. For the following comparison between Re-LT and LT patients, only the most recent survey was used. When the second survey was performed in 2013, the relatives of 27 patients informed us that the patient had deceased in the meantime.

Fifteen patients with a history of Re-LT could be identified (Re-LT group). There were 7 male (46.7%) and 8 female patients (53.3%) with a mean age of 53.6 years (standard deviation, SD = 8.4, range 31–66). The matched LT group (*n* = 60) consisted of 28 male (46.7%) and 32 female patients (53.3%), with a mean age of 53.5 years (SD = 8.8, range 24–66).

Of these 75 patients, 19 (25.3%) were interviewed during Survey 1 (in 2011), 33 (44%) were interviewed during both Survey 1 and Survey 2, and 23 (30.7%) were interviewed during Survey 2 in 2013.

Between both of the groups, there were no significant differences of the variables concerning gender, age, an underlying disease, religiousness, family status, relationship status, comorbidities, and the time elapsed since the transplantation (Re-LT group: last transplantation) (all *p* > 0.05, Table 1).

**Table 1:** Patient characteristics

		Re-LT	LT	
		n (%)/mean	n (%)/mean	p
Gender				1.000
	Male	7 (46.7%)	28 (46.7%)	
	Female	8 (53.3%)	32 (53.3%)	
	Total	15 (100%)	60 (100%)	
Age	(years)	53.6 (SD = 8.4)	53.5 (SD = 8.8)	0.958
	<55 years	8 (53.3%)	32 (53.3%)	1.000
	≥55 years	7 (46.7%)	28 (46.7%)	
Underlying disease				0.159
	Alcoholic liver cirrhosis	3 (20.0%)	31 (51.7%)	
	Cryptoenic liver cirrhosis	2 (13.3%)	5 (8.3%)	
	Cholestatic bile duct diseases	2 (13.3%)	5 (8.3%)	
	Viral hepatitis	3 (20.0%)	2 (3.3%)	
	Acute liver failure	1 (6.7%)	7 (11.7%)	
	Autoimmune hepatitis	1 (6.7%)	3 (5.0%)	
	Others	3 (20.0%)	7 (11.7%)	
Comorbidities				
	Arterial hypertension	9 (60.0%)	40 (66.7%)	0.763
	Diabetes mellitus	6 (40.0%)	21 (35.0%)	0.768
	Chronic kidney failure	12 (80.0%)	32 (53.3%)	0.081
Time since transplantation				0.594
	<5 years	10 (66.7%)	40 (66.7%)	
	5–10 years	4 (26.7%)	11 (18.3%)	
	>10 years	1 (6.7%)	9 (15.0%)	
Committed relationship				0.448
	Yes	10 (66.7%)	32 (53.3%)	
	No	1 (6.7%)	12 (20.0%)	
	No answer	4 (26.7%)	16 (26.7%)	
Family status				0.814
	Unmarried	1 (6.7%)	7 (11.7%)	
	Married	8 (53.3%)	31 (51.7%)	
	Divorced	1 (6.7%)	6 (10.0%)	
	Widowed	1 (6.7%)	1 (1.7%)	
	No answer	4 (26.7%)	15 (25.0%)	
Religious				0.448
	Yes	1 (6.7%)	10 (16.7%)	
	No	10 (66.7%)	35 (58.3%)	
	No answer	4 (26.7%)	15 (25.0%)	

*Re-LT* Liver retransplantation group, *LT* Liver transplantation group. Statistical test: Pearson Chi-Square

### SF-36 results

**Fig. 2:**
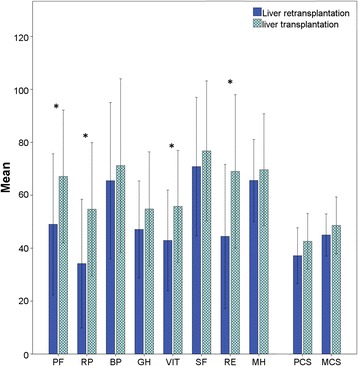
SF-36 results of both groups. Re-LT = Liver Retransplantation group, LT = Liver Transplantation group. PF = physical functioning, RP = role-physical, BP = bodily pain, GH = general health perceptions, VIT = Vitality, SF = social functioning, RE = role-emotional, MH = mental health, PCS = physical component summary, MCS = mental component summary. * Statistically significant

The HRQoL scores were lower in all 8 dimensions, as well as in the summation scales of PCS and MCS, among the Re-LT group when compared to the LT group. Statistically significant differences were found for PF (*p* = 0.026), RP (*p* = 0.008), VIT (*p =* 0.040), and RE (*p* = 0.005). Figure 2 shows the SF-36 results.

### HADS results

**Fig. 3:**
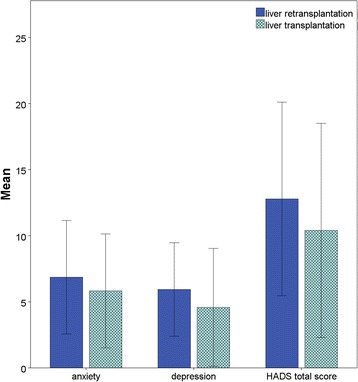
HADS results for both groups. Re-LT = Liver Retransplantation group, LT = Liver Transplantation group. * Statistically significant

The highest values were found in the Re-LT group for anxiety, depression, and the totals of the HADS scores. The differences were not statistically significant (*p =* 0.310, 0.123 and 0.213, Fig. 3).

### Impact of the clinical and demographical variables on the mental status and the HRQoL

In a second step, the impact of the comorbidities (chronic kidney disease, arterial hypertension, diabetes mellitus) as well as demographical factors such as age (<55 and ≥55 years, respectively) and gender on the HRQoL (Table 2) and mental status (Table 3) was assessed in a univariate analyis. With regard to the HADS scores and SF-36 scores in the Re-LT group and the LT group, the factors arterial hypertension, diabetes mellitus, gender and age did not have a significant impact (all *p* > 0.05). Those patients who suffered from a chronic kidney disease had decreased scores for PF when compared to the patients with a normal kidney function (*p* = 0.011, Table 2). In a multiple regression analysis on all HADS and SF-36 dimensions, chronic kidney disease was found to be the only independent risk factor for reduced PF scores (Correlation coefficient *B* = −0.279, *t* = −2.319, *p* = 0.023). All other variables did not show significances in the multiple regression analysis (all *p*-values >0.05).

**Table 2:** Univariate analysis: impact of clinical and demographical variables on SF-36 results of all patients (*n* = 75)

Clinical variable	SF-36 dimension															
	PF		RP		BP		GH		VIT		SF		RE		MH	
	M (SD)	p	M (SD)	p	M (SD)	p	M (SD)	p	M (SD)	p	M (SD)	p	M (SD)	p	M (SD)	p
Chronic kidney disease																
Yes	56.8 (26.5)	0.011	48.4 (27.0)	0.412	66.8 (32.8)	0.309	53.5 (22.3)	0.831	51.8 (22.9)	0.689	75.3 (28.2)	0.768	64.1 (31.0)	0.850	69.5 (21.2)	0.490
No	71.9 (23.4)		52.2 (24.3)		73.8 (31.3)		53.9 (19.9)		55.2 (19.6)		75.4 (24.7)		62.2 (29.7)		66.9 (19.6)	
Diabetes mellitus																
Yes	62.6 (26.8)	0.938	50.0 (28.9)	0.872	71.8 (30.3)	0.815	55.2 (20.7)	0.663	55.0 (21.3)	0.657	77.3 (28.6)	0.441	64.7 (32.3)	0.851	70.0 (18.2)	0.799
No	63.3 (26.2)		50.0 (24.3)		68.5 (33.4)		52.7 (21.7)		52.2 (21.8)		74.2 (25.7)		62.6 (29.4)		67.6 (21.7)	
Arterial hypertension																
Yes	60.9 (27.2)	0.349	49.6 (27.3)	0.792	65.8 (33.3)	0.138	52.6 (22.0)	0.467	52.4 (22.7)	0.746	74.5 (25.5)	0.475	61.3 (30.2)	0.414	65.6 (20.7)	0.097
No	67.1 (24.3)		50.7 (23.4)		76.8 (29.3)		55.6 (20.0)		54.7 (19.3)		76.9 (29.1)		67.9 (30.8)		73.8 (19.3)	
Age																
< 55 years	66.6 (27.2)	0.136	53.8 (28.0)	0.230	74.2 (323)	0.151	55.5 (21.5)	0.321	54.8 (19.2)	0.594	75.0 (28.9)	0.792	68.4 (32.4)	0.114	69.4 (18.8)	0.609
≥ 55 years	59.0 (24.8)		45.7 (22.8)		64.6 (31.7)		51.4 (21.0)		51.4 (24.1)		75.7 (24.2)		57.6 (27.0)		67.3 (22.5)	
Gender																
Male	64.3 (26.3)	0.733	51.3 (27.4)	0.707	65.9 (33.4)	0.306	53.2 (22.8)	0.786	52.5 (22.1)	.737	76.8 (24.8)	0.934	61.0 (29.4)	0.636	70.3 (19.7)	0.465
Female	61.7 (26.4)		48.6 (24.3)		73.9 (30.7)		54.2 (19.8)		54.0 (21.2)		74.1 (28.5)		654 (31.3)		66.8 (21.2)	

*SF-36* Short Form 36, *PF* Physical Function, *RP* Role Physical, *BP* Bodily Pain, *GH* General Health, *VIT* Vitality, *SF* Social Function, *RE* Role emotional, *MH* Mental Health, *M* Mean, *SD* Standard Deviation. Statistical test: Man Whitney U test

**Table 3:** Univariate analysis: Impact of clinical and demographical variables on Hospital Anxiety and Depression Scale results of all patients (*n* = 75)

	Hospital Anxiety and Depression Scale dimensions			
Clinical variable	Anxiety		Depression	
	M (SD)	p	M (SD)	p
Chronic kidney disease				
Yes	5.4 (3.7)	0.265	5.0 (4.2)	0.574
No	7.0 (5.0)		4.6 (4.6)	
Diabetes mellitus				
Yes	6.3 (4.0)	0.557	4.5 (3.4)	0.943
No	5.9 (4.5)		5.0 (4.8)	
Arterial hypertension				
Yes	6.7 (4.4)	0.075	5.5 (4.7)	0.225
No	4.9 (4.0)		3.8 (3.2)	
Age				
< 55 years	6.1 (4.7)	0.628	4.9 (4.5)	0.096
≥ 55 years	5.9 (3.9)		4.8 (4.1)	
Gender				
Male	6.0 (4.9)	0.945	4.0 (3.8)	0.986
Female	6.1 (3.6)		5.7 (4.7)	

*M* Mean, *SD* Standard Deviation. Statistical test: Man Whitney U test

## Discussion

The present study has shown that the Re-LT patients had significantly impaired HRQoL scores, with regards to their physical dimensions. Their mental status, including the anxiety and depression scores, did not differ significantly between the groups. We have shown that those patients who were suffering from a chronic kidney failure had decreased physical function scores, whereas the underlying liver disease had no impact on the results of both questionnaires.

Our group has previously reported good HRQoL and mental status scores among LT recipients when compared to the patients on the LT waiting list and the general population, respectively [7, 8]. In the current literature, there are numerous studies analysing the psychosocial factors in LT recipients [7, 15, 17, 18].

With regards to the results of the HADS survey, the anxiety and depression scores were lower than those in previous studies [7, 23, 24]. We have previously shown that the mental status (anxiety) of patients who underwent an LT was affected when compared to the general population [16]. Although not statistically significant, patients from the Re-LT group tended to have higher anxiety and depression scores, so there seems to be a further decrease in mental status after Re-LT. Since there are a few studies examining HRQoL but not mental status after Re-LT [9, 12, 13], the interpretation of our data in a greater context is difficult and speculative. Re-LT is associated with a higher rate of chronic graft failure [25]. Patients on the liver transplantation waiting list have who suffer from chronic liver disease were found to have higher levels of anxiety and depression when compared to LT recipients [8]. Some HRQoL studies include Re-LT patients [9, 12–14]. However, in these examinations, there is no thorough analysis of this subgroup. Braun and colleagues mention a significant loss of HRQoL among Re-LT patients without further specification [13]. These findings are in line with our results. Although there is a considerable number of Re-LT patients each year, this patient group is completely underrepresented in HRQoL analyses. In most cases, a graft loss is due to a primary nonfunction or vascular thrombosis. About 8% of all LT recipients need to undergo a retransplantation [26, 27]. Clinical data has shown that both patient [26, 28, 29] and graft survival [2] are significantly reduced in Re-LT patients when compared to primary LT recipients. Re-LT is associated with an increased postoperative morbidity, including primary graft dysfunction, adult respiratory distress syndrome, infections, and acute renal failures, when compared to a primary LT [15]. This might be an explanation for the fact that there was a significant decrement in the HRQoL dimensions of PF, RP, VIT, and RE. An important aspect is the longevity of an LT, which is associated with a higher HRQoL [9]. For various reasons, such as the difficulties of redo surgery procedures, immunological problems after repeated organ transplantations, and a general poor health status at the time of the retransplantation, long-term graft survival is significantly impaired after an Re-LT [26, 30].

Montenevo have analysed a large cohort of Re-LT recipients and found that these patients were more likely to be on dialysis prior to transplant, hospitalized, in the intensive care unit, on a ventilator and had higher model for end-stage liver disease (MELD) while they had, on the other hand, lower Donor Risk scores when compared to primary LT recipients [6]. The authors state that higher quality grafts are used inefficiently in a sicker patient population and propose a more optimal strategy including restricting their use to patients who obtain a longer term benefit [6]. Although we have not analyzed the clinical course of the patients examined in the present study, the decrement, especially in HRQoL scores of Re-LT patients compared to primary LT patients underlines the need for an optimized organ distribution strategy. Our analysis further revealed that those patients who suffered from chronic kidney disease had lower PF scores compared to patient who patients who had not. These findings are in line with the current literature, HRQoL was found to be decreased in renal patients in the early stages of disease [31].

The comparison of our results with other studies is somewhat difficult, since there are no HRQoL studies on Re-LT patients. Nonetheless, our findings have indicated that patients who undergo an Re-LT, are at a risk of suffering from psychological disorders, as well as from an impaired HRQoL. As a consequence, a profound selection process of patients who are scheduled for an Re-LT has to be performed. One of the shortcomings is the cross-sectional design of this study. Additionally, the number of Re-LT patients was small. Since the response rate of the postal survey was 40%, there might be some selection bias which could possibly lead to the fact that only patients with either a extremely high or low HRQoL respond to the survey. Moreover, the interpretation of data is limited by the lack of clinical data such as performance status, the presence of ascites and icterus, and laboratory data since these factors are known to have an impact on physical status. Furthermore, the HRQoL data analysed in this examination are from two different surveys using different assessment methods. Since, it is known that different modes of HRQoL assessments lead to differing results [32]. Also, there is some potential time effect, since, as far as the group that was interviewed twice is concerned, only the most recent surveys (2013) were used for the comparisons. HRQoL might change over time and with increasing age, so this is a potential bias to our data. Although there were no statistically significant differences between the two assessments, these effects cannot completely be excluded. However, recent studies have only included Re-LT patients as a part of a bigger study collective without a closer examination of this subgroup. The present study is the first study to thoroughly focus on the HRQoL and the mental status in Re-LT patients. Moreover, we have established a matched control group, allowing for a good comparison of the results. Nevertheless, there is an urgent need for further studies, with larger sample sizes, in order to draw final conclusions on this topic.

## Conclusion

Patients who have to undergo Re-LT procedures are facing a postoperative impairment, especially with the physical aspects of a HRQoL. Together with clinical results from other studies, the findings of the present examination underline the need for an optimized organ distribution strategy since not all patients listed for Re-LT appear to benefit from it.

## Abbreviations

AH: Arterial hypertension
ALF: Acute liver failure
CKF: Chronic kidney failure
DM: Diabetes mellitus
ESLD: End-stage liver disease
GH: General health
HADS: Hospital Anxiety and Depression Scale
HRQoL: Health-related quality of life
ITBL: Ischemic-type biliary lesions
LT: Liver transplantation
MCS: Mental component summary
MH: Mental health
PCS: Physical component summary
PF: Physical function
PHQ-4: Patient health questionnaire-4
RE: Role emotional
Re-LT: Liver retransplantation
RP: Role physical
SD: Standard deviation
SF: Social function
SF-36: Short form-36
VIT: Vitality
